# A Rare Case of Ketosis-Prone Type 2 Diabetes With a Unique Human Leukocyte Antigen (HLA) Profile: Genetic and Metabolic Insights

**DOI:** 10.7759/cureus.77247

**Published:** 2025-01-10

**Authors:** Mai Mukai, Nobuyuki Koriyama, Ryotaro Hirahara, Kanako Wada, Yoshihiko Nishio

**Affiliations:** 1 Diabetes and Endocrine Medicine, National Hospital Organization (NHO) Kagoshima Medical Center, Kagoshima, JPN; 2 Diabetes and Endocrine Medicine, Kagoshima University, Kagoshima, JPN

**Keywords:** diabetic ketoacidosis, hla gene haplotype, ketosis-prone diabetes, type 1 diabetes, type 2 diabetes

## Abstract

This report describes the case of a 21-year-old man with ketosis-prone type 2 diabetes (KPD), highlighting the genetic and metabolic factors influencing disease progression. The patient presented with diabetic ketoacidosis and demonstrated rapid insulin secretory recovery, allowing early discontinuation of insulin. Genetic analysis revealed human leukocyte antigen (HLA)-DRB1*15:01 and DRB1*09:01 haplotypes, suggesting a role of immune factors typically associated with type 1 diabetes in the pathogenesis of KPD. Metabolic factors, evidenced by prolonged ketone clearance, further underscore the complexity of KPD. These findings contribute to a growing understanding of KPD as a unique subtype of diabetes, reinforcing the importance of personalized, genetically informed management approaches for optimal care.

## Introduction

In 1987, Winter et al. offered the first description of young, obese individuals with type 2 diabetes (T2D) who developed diabetic ketoacidosis (DKA) without apparent triggers [1]. These individuals experienced recurrent episodes of DKA or ketosis but showed marked recovery of insulin secretion within a few months, allowing insulin withdrawal. This atypical form of T2D, termed ketosis-prone T2D (KPD), was initially thought to be prevalent mainly among African-American populations [2], but recent studies have increasingly reported cases in Asian populations [3]. KPD is predominantly observed in young, obese men with a family history of diabetes [4]. The pathology is often triggered by stress [5] or weight gain [6] and develops suddenly with ketosis or ketoacidosis [6]. It is inferred that understanding and responding to the characteristics of KPD is crucial for improving long-term glycemic control status and managing the risk of complications, while education based on these characteristics will also contribute to improving patient self-management skills. Genetic studies have shown a distinct human leukocyte antigen (HLA) profile for KPD, with a significantly lower prevalence of the type 1 diabetes (T1D)-susceptible haplotype HLA-DRB1*09:01 and a higher prevalence of the protective haplotypes HLA-DRB1*15:01 and/or HLA-DRB1*15:02 [7]. Here, we present a rare case of KPD with both HLA-DRB1*09:01 and HLA-DRB1*15:01.

## Case presentation

A 21-year-old man presented to a family doctor with a two-week history of thirst, polydipsia, polyuria, and significant weight loss (9 kg). He had a history of ureteral stones and a family history of T2D; his father, who had T2D, had died of myocardial infarction. The patient presented with a blood glucose level of 460 mg/dL and ketonuria (4+), prompting referral to our department. On admission, DKA was confirmed based on a glycohemoglobin A1c (HbA1c) level of 14.0% and a serum total ketone level of 10,101 μmol/L. Arterial blood gas analysis showed a pH of 7.296, arterial partial pressure of carbon dioxide at 28 mmHg, arterial partial pressure of oxygen at 104.8 mmHg, bicarbonate ions (HCO3-) at 13.4 mmol/L, base excess of -11.1 mmol/L, and anion gap of 24.90 mmol/L, indicating metabolic acidosis with a high anion gap and respiratory compensation (Table 1).

**Table 1:** Laboratory findings WBC: white blood cells, RBC: red blood cells, Hb: hemoglobin, PLT: platelets, AST: aspartate aminotransferase, ALT: alanine aminotransferase, LDH: lactate dehydrogenase, ALP: alkaline phosphatase, γ-GTP: γ-glutamyltransferase, T.Bil: total bilirubin, chE: cholinesterase, AMY: amylase, CK: creatine kinase, Alb: albumin, Na: sodium, K: potassium, Cl: chloride, Ca: calcium, IP: inorganic phosphorus, Mg: magnesium, BUN: blood urea nitrogen, Cr: creatinine, eGFR: estimated glomerular filtration rate, UA: uric acid, CRP: C-reactive protein, TG: triglycerides, LDL: low-density lipoprotein cholesterol, HDL: high-density lipoprotein cholesterol, PaCO2: arterial partial pressure of carbon dioxide, PaO2: arterial partial pressure of oxygen, HCO3-: bicarbonate ion, BE: base excess, AG: anion gap

	Laboratory test	Value		Reference range
Urinalysis	Protein	(+)	-	-
	Glucose	(4+)	-	-
	Ketone bodies	(4+)	-	-
Peripheral blood	WBC	8240	/μL	3300-8600
	RBC	595	×10^4^/μL	435-555
	Hb	17.5	g/dL	13.7-16.8
	PLT	333	×10^4^/μL	158-348
Biochemistry	AST	32	U/L	13-30
	ALT	73	U/L	10-42
	LDH	173	U/L	124-222
	ALP	117	U/L	38-113
	γ-GTP	65	U/L	13-64
	T. Bil	0.56	mg/dL	0.40-1.50
	chE	553	U/L	240-486
	AMY	69	mg/dL	44-132
	CK	105	U/L	59-248
	Alb	5.03	g/dL	4.10-5.10
	Na	136	mmol/L	138-145
	K	4	mmol/L	3.6-4.8
	Cl	98	mmol/L	101-108
	Ca	9.7	mg/dL	8.8-10.1
	IP	2.7	mg/dL	2.7-4.6
	Mg	2.1	mg/dL	1.8-2.3
	BUN	10.7	mg/dL	8.0-20.0
	Cr	0.7	mg/dL	0.65-1.07
	eGFR	113.8	mL/min/1.73 m^2^	>60
	UA	8.9	mg/dL	3.7-7.8
	CRP	0.18	mg/dL	0.00-0.14
	TG	207	mg/dL	0.00-0.14
	LDL	218	mg/dL	65-163
	HDL	36	mg/dL	38-90
Arterial blood gas	pH	7.296	-	7.350-7.450
	PaCO_2_	28.1	mmHg	35.0-45.0
	PaO_2_	104.8	mmHg	83.0-108.0
	HCO_3_^-^	13.4	mmol/L	22.0-28.0
	BE	-11.1	mmol/L	-2 - +2
	AG	24.9	mmol/L	10.0-14.0

Height was 167.8 cm, weight was 70.6 kg, body mass index (BMI) was 25.1 kg/m², blood pressure was 148/82 mmHg, and heart rate was 122 beats/min. Body temperature was 37.1°C, respiratory rate was 16 breaths/min, and peripheral oxygen saturation on pulse oximetry was 97.0%. No other physical abnormalities or complications were identified.

Fasting serum C-peptide immunoreactivity (CPR) (2.19 ng/mL) and a CPR index (CPI: calculated as fasting serum CPR/fasting plasma glucose × 100) of 0.6 indicated significantly reduced endogenous insulin secretion. Results for islet autoantibodies were negative (Table 2).

**Table 2:** Endocrinological laboratory findings GADA: anti-glutamic acid decarboxylase antibody, IA-2A: anti-insulinoma-associated protein-2 antibody, IAA: anti-insulin autoantibody, ICA: islet cell antibody, ZnT8A: zinc transporter 8 antibody, TgA: anti-thyroglobulin antibody, TPOA: anti-thyroperoxidase antibody, FT4: free thyroxine, TSH: thyroid-stimulating hormone, FPG: fasting plasma glucose, HbA1c: glycohemoglobin A1c, IRI: immunoreactive insulin, CPR: C-peptide immunoreactivity, CPI: CPR index, 24-h UCPR: 24-h urinary CPR, TK: total ketone bodies, 3HBA: 3-hydroxybutyric acid, AA: acetoacetic acid ΔCPR: calculated by subtracting pre-load CPR from post-load CPR; GADA, IA2A, and ZnT8: measured by enzyme-linked immunosorbent assay; ICA: measured by an indirect method with immunofluorescent antibody; TgA and TPOA: measured by electrochemiluminescent immunoassay

Laboratory test	Value		Reference range
GADA	<5.0	U/mL	<5.0
IA-2A	<0.6	U/mL	<0.6
IAA	<0.4	U/mL	<0.4
ICA	Negative (<1.25)	JDF units	Negative (<1.25)
ZnT8A	<10.0	U/mL	<10.0
TgA	<10.0	IU/mL	<19.0
TPOA	2.5	IU/mL	<3.0
FT4	0.98	ng/dL	0.78-1.48
TSH	1.021	μU/mL	0.610-4.230
FPG at onset	357	mg/dL	73-109
HbA1c at onset	14.3	%	4.9-6.0
IRI at onset	6.3	μU/mL	5.0-10.0
Fasting CPR at onset	2.19	ng/mL	0.61-2.09
Fasting CPI at onset	0.6	-	1.03-2.17
24-h UCPR at onset	18.7	μg/day	29.2-167.0
TK at onset	10101	μmol/L	<130
3HBA at onset	7602	μmol/L	<85
AA at onset	2499	μmol/L	<55
Fasting CPR on day 8	2	ng/mL	0.61-2.09
Fasting CPI on day 8	1.6	-	1.03-2.17
24-h UCPR on day 12	70.2	μg/day	29.2-167.0
ΔCPR in glucagon tolerance test on day 15	1.82	ng/mL	>1.0

The HLA haplotypes were A*02:01, DRB1*09:01-DQB1*03:03, and DRB1*15:01-DQB1*06:02. A*02:0 has been reported to be involved in pancreatic β-cell dysfunction7. DRB1*09:01-DQB1*03:03 is reportedly associated with T1D susceptibility, whereas DRB1*15:01-DQB1*06:02 is reportedly associated with T1D resistance. These findings suggested the presence of a unique KPD profile-related immune gene (Table 3).

**Table 3:** HLA genotyping HLA: human leukocyte antigen

	Allele	Allele
A	02:01:01	26:03:01
B	15:01:01	39:01
C	03:03:01	07:02:01
DRB1	09:01:02	15:01
	DR9	DR15
DPB1	02:01:02	-
	or	
	02:01:02	141:01
	or	
	141:01	-
DQB1	03:03:02	06:02:01

**Figure 1:**
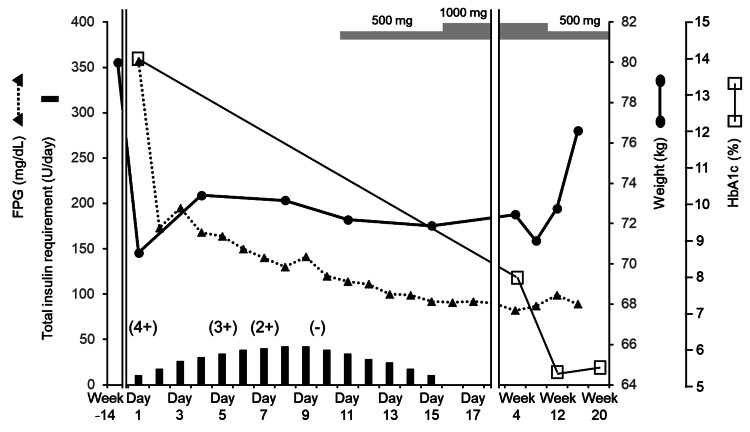
Clinical course of the patient The bar graph at the bottom depicts the total daily insulin requirement. The dashed line with closed triangles indicates fasting blood glucose levels, while the thick solid line with closed circles shows body weight. The thin solid line with open squares denotes HbA1c levels. Values within parentheses with "+" or "-" signs indicate changes in urinary ketone levels over time. Gray-shaded bars at the top represent the dosage of metformin administered during the observation period. HbA1c: glycohemoglobin A1c

The patient was treated with insulin lispro and degludec, along with saline infusion, transitioning to dextrose by day 2. Insulin requirements peaked on days 8 and 9 and were tapered as metformin was introduced on day 11. During this period, blood glucose levels remained within 140-270 mg/dL (Figure 1), and urinary ketones became negative on day 10. On day 16, insulin was discontinued, and the patient was discharged. Four weeks later, HbA1c had improved to 8.0% with a lower metformin dose, stabilizing at 5% by week 8 (Figure 1). CPI on day 8 was 1.6, urinary CPR on day 12 had significantly recovered from 18.7 to 70.2 μg/day, and ΔCPR in the glucagon tolerance test performed on day 15 was 1.82 ng/mL (Table 2).

## Discussion

The patient was a young, obese male with a family history of T2D on the paternal side, and hyperglycemia was initially noted at the onset of DKA. Insulin secretory capacity recovered quickly after initiating treatment, allowing early discontinuation of insulin therapy. Given these characteristics, KPD was diagnosed. In cases initially resembling T1D, endogenous insulin secretion can sometimes improve, so considering the possibility of KPD is important in such presentations. According to some reports, KPD often recurs within two years after remission, with biguanides being effective in extending the remission period [4]. Consequently, metformin therapy was initiated for this patient, who achieved good glycemic control with an HbA1c of 5.3% at 12 weeks (Figure 1). However, repeated episodes of DKA may lower the body weight threshold for onset and progressively impair insulin secretion. Therefore, weight gain trends in this patient will need to be monitored closely (Figure 1).

KPD can be classified into four categories based on different diagnostic criteria: the American Diabetes Association (ADA) classification, the modified ADA classification, the BMI classification, and the Aβ classification [8]. The Aβ classification system divides patients into four subgroups according to the presence or absence of islet autoantibodies and β-cell function: antibody present with function absent (A+β-), antibody present with function present (A+β+), antibody absent with function absent (A-β-), and antibody absent with function present (A-β+) [8]. This classification system has clinical relevance, providing insights into glycemic control and insulin dependency status, thus serving as a valuable tool in both clinical and research settings. The Aβ classification of this case was A-β+.

Interestingly, Satomura et al. reported that approximately 36.4% of A-β+ KPD cases exhibit specific immunoreactivity to the insulin B chain amino acids 9-23-related peptide (B:9-23rPep), which is associated with interferon γ and reflects disease activity in T1D [7]. Detection rates of this immunoreactivity have been reported as 38.1% in acute-onset T1D and 8% in T2D, suggesting a potential role in the pathogenesis of T1D [7]. These findings and reports indicating reduced insulin secretion over time in KPD (A-β+) [6] support the hypothesis that KPD may represent a subtype of T1D with overlapping features.

In Japanese populations, the typical T1D susceptibility haplotypes are DRB1*0405-DQB1*0401, DRB1*0901-DQB1*0303, and DRB1*0802-DQB1*0302, while the typical resistance haplotypes are DRB1*1501-DQB1*0602 and DRB1*1502-DQB1*0601 [9]. Resistant alleles are known to be dominant over susceptibility alleles with respect to the development of T1D [10]. Genetically, about 60% of KPD patients possess HLA-DR3 or HLA-DR4 haplotypes associated with T1D susceptibility [11]. Approximately 30% show DRB1*15:01 or DRB1*15:02, representing HLA haplotypes associated with T1D resistance, and 50% carry A*02:01 or A24:02, which are linked to pancreatic β-cell dysfunction. The genotypes in this patient included HLA-DRB1*15:01 and DRB1*09:01, a T1D susceptibility haplotype, and A*02:01 (Table 2). Although the pathogenesis of KPD remains unclear, this genetic information suggests that both the genetic background and immune response mechanisms seen in T1D could also be involved in KPD onset and activity. Major histocompatibility complex II of the T1D resistance haplotype reportedly presents islet antigens (suppressive antigens) to regulatory T cells (Tregs), thereby promoting the production of anti-inflammatory cytokines such as interleukin 10 and suppressing the activity of autoreactive islet-responsive T cells [12]. The T1D resistance haplotype is speculated to contribute to pancreatic β-cell protection in KPD by promoting specific Treg activation and suppressing autoreactive T cells. Further research would be extremely beneficial in elucidating the cellular pathways that promote the onset and progression of organ-specific autoimmune diseases such as T1D or clarifying how to induce tolerance.

In KPD, (1) leucine catabolism is enhanced; (2) α-glutamate accumulates due to a kinetic defect in ketoglutarate dehydrogenase; (3) anaplerosis of glucose carbon for the tricarboxylic acid (TCA) cycle is blunted; and (4) impaired peripheral oxidation of ketone bodies, which depends on the TCA cycle, has been reported [13]. In this case, the relatively prolonged duration of 10 days required for ketone clearance suggests a role of metabolic factors in the pathophysiology of KPD (Figure 1). Other reports have linked KPD to glucose toxicity [14], reduced glucose-6-phosphate dehydrogenase activity [15], and inadequate glucagon inhibition following glucose loading [16]. These observations suggest a complex metabolic profile for KPD, likely involving genetic abnormalities in metabolism-related enzymes. Further research is needed to clarify these pathways' roles and identify potential therapeutic targets.

## Conclusions

This case highlights the unique genetic and metabolic characteristics of KPD, contributing to our understanding of its pathogenesis and clinical management. The rapid recovery of insulin secretion and subsequent discontinuation of insulin in this patient, alongside his specific HLA haplotypes (HLA-DRB1*15:01 and DRB1*09:01), underscore the potential influence of genetic background and immune response on KPD. In addition, the extended duration required for ketone clearance suggests an underlying metabolic complexity, possibly related to abnormalities in ketone metabolism and amino acid pathways. These findings reinforce the notion that KPD may represent a distinct subtype of diabetes, potentially overlapping with T1D in terms of genetic and immunological profiles. Ongoing genetic and immunological studies are essential to fully elucidate the mechanisms underlying KPD and to inform targeted therapeutic strategies. Further, longitudinal monitoring of glycemic control and weight gain is crucial, as recurrence is common, and weight fluctuations may affect insulin secretion capacity over time. This case adds valuable insights into the nuanced management of KPD, emphasizing a need for tailored therapeutic approaches in light of complex interplays between genetic, immunologic, and metabolic factors.

## References

[1] Winter WE, Maclaren NK, Riley WJ, Clarke DW, Kappy MS, Spillar RP (1987). Maturity-onset diabetes of youth in black Americans. N Engl J Med.

[2] Vellanki P, Umpierrez GE (2017). Diabetic ketoacidosis: a common debut of diabetes among African Americans with type 2 diabetes. Endocr Pract.

[3] Gupta RD, Ramachandran R, Gangadhara P (2017). Clinical characteristics, beta-cell dysfunction and treatment outcomes in patients with A-β+ Ketosis-Prone Diabetes (KPD): The first identified cohort amongst Asian Indians. J Diabetes Complications.

[4] Umpierrez GE, Smiley D, Kitabchi AE (2006). Narrative review: ketosis-prone type 2 diabetes mellitus. Ann Intern Med.

[5] Small C, Egan AM, Elhadi EM, O'Reilly MW, Cunningham A, Finucane FM (2017). Diabetic ketoacidosis: a challenging diabetes phenotype. Endocrinol Diabetes Metab Case Rep.

[6] Satomura A, Oikawa Y, Haisa A, Inoue I, Noda M, Shimada A (2020). Bodyweight threshold for sudden onset of ketosis might exist in ketosis-prone type 2 diabetes patients. J Diabetes Investig.

[7] Satomura A, Oikawa Y, Haisa A, Suzuki S, Nakanishi S, Katsuki T, Shimada A (2022). Clinical significance of insulin peptide-specific interferon-γ-related immune responses in ketosis-prone type 2 diabetes. J Clin Endocrinol Metab.

[8] Maldonado M, Hampe CS, Gaur LK (2003). Ketosis-prone diabetes: dissection of a heterogeneous syndrome using an immunogenetic and beta-cell functional classification, prospective analysis, and clinical outcomes. J Clin Endocrinol Metab.

[9] Kawabata Y, Ikegami H, Kawaguchi Y (2002). Asian-specific HLA haplotypes reveal heterogeneity of the contribution of HLA-DR and -DQ haplotypes to susceptibility to type 1 diabetes. Diabetes.

[10] Kawabata Y, Ikegami H, Awata T (2009). Differential association of HLA with three subtypes of type 1 diabetes: fulminant, slowly progressive and acute-onset. Diabetologia.

[11] Banerji MA, Chaiken RL, Huey H (1994). GAD antibody negative NIDDM in adult black subjects with diabetic ketoacidosis and increased frequency of human leukocyte antigen DR3 and DR4. Flatbush diabetes. Diabetes.

[12] Choukem SP, Sobngwi E, Boudou P (2013). β- and α-cell dysfunctions in africans with ketosis-prone atypical diabetes during near-normoglycemic remission. Diabetes Care.

[13] Mitchell AM, Michels AW (2022). Self-antigens targeted by regulatory T cells in type 1 diabetes. Int J Mol Sci.

[14] Patel SG, Hsu JW, Jahoor F (2013). Pathogenesis of A⁻β⁺ ketosis-prone diabetes. Diabetes.

[15] Gosmanov AR, Smiley D, Robalino G, Siqueira JM, Peng L, Kitabchi AE, Umpierrez GE (2010). Effects of intravenous glucose load on insulin secretion in patients with ketosis-prone diabetes during near-normoglycemia remission. Diabetes Care.

[16] Sobngwi E, Gautier JF, Kevorkian JP (2005). High prevalence of glucose-6-phosphate dehydrogenase deficiency without gene mutation suggests a novel genetic mechanism predisposing to ketosis-prone diabetes. J Clin Endocrinol Metab.

